# Production of Thermoalkaliphilic Lipase from *Geobacillus thermoleovorans* DA2 and Application in Leather Industry

**DOI:** 10.1155/2016/9034364

**Published:** 2016-01-03

**Authors:** Deyaa M. Abol Fotouh, Reda A. Bayoumi, Mohamed A. Hassan

**Affiliations:** ^1^Electronic Materials Research Department, Advanced Technology and New Materials Institute (ATNMRI), City of Scientific Research and Technological Applications (SRTA-City), New Borg El-Arab City, P.O. Box 21934, Alexandria, Egypt; ^2^Biology Department, Faculty of Science and Education, Taif University, Khormah Branch, P.O. Box 21974, Taif, Saudi Arabia; ^3^Botany and Microbiology Department, Faculty of Science (Boys), Al-Azhar University, P.O. Box 11884, Cairo, Egypt; ^4^Protein Research Department, Genetic Engineering and Biotechnology Research Institute (GEBRI), City of Scientific Research and Technological Applications (SRTA-City), New Borg El-Arab City, P.O. Box 21934, Alexandria, Egypt

## Abstract

Thermophilic and alkaliphilic lipases are meeting a growing global attention as their increased importance in several industrial fields. Over 23 bacterial strains, novel strain with high lipolytic activity was isolated from Southern Sinai, Egypt, and it was identified as* Geobacillus thermoleovorans* DA2 using 16S rRNA as well as morphological and biochemical features. The lipase was produced in presence of fatty restaurant wastes as an inducing substrate. The optimized conditions for lipase production were recorded to be temperature 60°C, pH 10, and incubation time for 48 hrs. Enzymatic production increased when the organism was grown in a medium containing galactose as carbon source and ammonium phosphate as nitrogen source at concentrations of 1 and 0.5% (w/v), respectively. Moreover, the optimum conditions for lipase production such as substrate concentration, inoculum size, and agitation rate were found to be 10% (w/v), 4% (v/v), and 120 rpm, respectively. The TA lipase with Triton X-100 had the best degreasing agent by lowering the total lipid content to 2.6% as compared to kerosene (7.5%) or the sole crude enzyme (8.9%). It can be concluded that the chemical leather process can be substituted with TA lipase for boosting the quality of leather and reducing the environmental hazards.

## 1. Introduction

Lipases (triacylglycerol acylhydrolases, E.C. 3.1.1.3) are ubiquitous enzymes of considerable physiological significance and industrial potential [1]. Lipases catalyze the hydrolysis of triacylglycerols to glycerol and free fatty acids. Today, lipases are the choice of biocatalyst as they show unique chemo-, regio-, enantioselectivities, which enable the production of novel drugs, agrochemicals, and fine products [2].

Due to the ability of many lipases to perform both hydrolytic and synthetic reactions, they find immense applications in industries like foods, detergents, pharmaceuticals, leather, cosmetics, textile, dairy, and even biodiesel [3, 4].

Lipases are widely present in plants and animals, but almost all the commercially available lipases are usually obtained from microorganisms that produce a wide variety of extracellular lipases [5].

Cost of lipase production process was considered as a major obstacle in the industries. Therefore, many efforts are being made to use wastes as raw materials for lipase production. Agricultural residues for lipase production as well as other value added products would hold a prominent position in future biotechnologies, mainly because of its ecofriendliness and flexibility to both developing and developed countries. Several residues such as oil cakes, fibrous residues, and industrial effluent have increasing attention as abundant and cheap renewable feedstock [5, 6]. Enzymes from thermophiles and alkaliphiles have become the subject of special interest for biotechnological applications due to their high stability at adverse operational and/or storage conditions [7].

Many advantages were earned for carrying out biotechnological and industrial processes in high temperatures: high solubility of substrates (in particular for poorly soluble or polymeric molecules) resulting in higher product yield, higher reaction rates, increased availability of substrates, decreased risk of microbial contamination, and lower viscosity of reaction mixtures which in turn reduces the costs related to pumping, filtration, and centrifugation, and saving a great power cost would be exploited for cooling [8, 9].

In the present study, a detailed description of isolation, identification, and optimization of TA lipase production conditions from* G. thermoleovorans* DA2 will be demonstrated. An attempt to utilize restaurant fatty wastes as the main substrate for lipase production was carried out, which may raise the lipolytic activity and decrease the overall cost of the production process. In addition, this approach has a great environmental endeavor through minimizing the ecological hazards accompanied with the accumulation of wastes.

The application of TA lipase from* G. thermoleovorans* DA2 in leather tanning process as a degreasing agent replaced the commonly utilized organic solvent (Kerosene). Kanagaraj et al. reported that it is fundamental to add hydrolytic enzymes such as lipases and proteases in the soaking step for helping the fat degradation and raise the leather quality [10].

Substitution of the traditional chemical tanning processing with more ecofriendly treating procedures, for example, the enzymatic steps, became a necessity because of the recorded environmental hazards resulting from the pollution of water, careless disposal of solid wastes, and gaseous emissions [11].

## 2. Materials and Methods

### 2.1. Bacterial Strain

A wide variety of samples were collected from many localities in Egypt, including desert and hot springs of Southern Sinai, Wadi El-Natron swamps, desert of Qina and Suez governorates, and the soil of El-Basateen slaughter house.

All samples were suspended in sterilized saline solution (0.85% w/v) which were cultured on plates of nutrient agar medium with pH 9 and incubated at 65°C for 48 hrs. The separated colonies had undergone a series of (agar streak method) for purification, and the morphological characteristics of each isolate were investigated. The purpose of this method was to isolate the thermoalkalophilic microorganisms.

### 2.2. Screening

#### 2.2.1. Qualitative Assay

All strains were screened for investigating their lipase activity on agar plates containing Rhodamine B 0.001% (w/v), nutrient broth 0.8% (w/v), NaCl 0.4% (w/v), agar 1% (w/v), and olive oil 3%, in distilled water and the pH was adjusted to be 9 [12].

Plates were incubated at 65°C for 18 hrs and the lipase activity was identified as an orange halo zone around colonies under UV light at 350 nm.

#### 2.2.2. Quantitative Assay

To detect the most potent thermoalkaliphilic lipase producing bacteria, all bacterial isolates were grown on medium composed of yeast extract 1 g; olive oil 10 mL; gum Arabic 10 g; CaCl_2_ 1 g; and mineral salt solution 1 mL per liter. The medium pH is initially adjusted at 9 by using 6 N NaOH and incubated at 65°C and 100 rpm for 18 hrs. The most potent thermoalkaliphilic lipase producing isolate was purified by “agar streak method” and underwent biochemical investigations and it was identified by 16S rRNA technique.

#### 2.2.3. Bacterial Identification

The bacterial isolate was identified using 16S rRNA sequence. The genomic DNA was extracted by the following method which was described by Sambrook et al. [13].

The PCR amplification was carried out according to Hassan et al. and Abdou and Hassan. The reaction was performed using forwarded 16S rRNA primer (5′-AAATGGAGGAAGGTGGGGAT-3′) and reverse 16S rRNA primer (5′-AGGAGGTGATCCAACCGCA-3′). The PCR machine (TECHNE TC-3000, FTC3/02) was programmed as follows: 3 min denaturation at 95°C, followed by 35 cycles that consisted of 1 min at 95°C, 1 min at 58°C, and 1 min at 72°C and the final extension was 10 min at 72°C [14, 15].

The PCR product was cleaned up for sequencing using Qiagen kit for DNA purification from aqueous PCR. DNA sequencing method which was developed by Sanger et al. was carried out using 3130X DNA Sequencer (Genetic Analyzer, Applied Biosystems, Hitachi, Japan) [16].

#### 2.2.4. Sequence Analysis and Phylogenetic Tree Construction

Similarity of the obtained nucleotide sequence was performed by basic local alignment search tool (BLAST) against reference sequences available in National Center for Biotechnology Information GenBank (NCBI GenBank). The reference sequences were collected from GenBank and the alignments using Clastal W were performed for constructing the phylogenetic tree using MEGA 5 software version 5.1 [17].

### 2.3. Lipase Production

*G. thermoleovorans* DA2 was grown on a liquid production medium containing (%, w/v) yeast extract, 0.1 g; NaNO_3_, 0.2 g; KH_2_PO_4_, 0.1 g; MgSO_4_·7H_2_O, 0.05 g; KCl, 0.05 g; and CaCl_2_, 0.1 g, supplemented with 1 g of fatty restaurant wastes.

The medium was adjusted at pH 9 (pH-Meter Model-420A Orion Co., USA) and 100 mL of medium in 500 mL Erlenmeyer flasks was inoculated with* G. thermoleovorans* DA2 and incubated at 65°C and 100 rpm for 18 hrs on a rotary shaker (Innova J-25 New Brunswick scientific, USA).

### 2.4. Assay of Thermoalkaliphilic Lipase Activity

Lipase activity was detected by a spectrophotometric assay using* p*-nitrophenyl laurate (*p*NPL) as a substrate according to Castro-Ochoa et al. and Amara et al. with slight modifications. In brief, the reaction mixture consisted of 0.1 mL enzyme extract, 0.8 mL 0.1 M phosphate buffer (pH 8), and 0.1 mL 0.01 M* p*NPL in isopropanol. The hydrolytic reaction was carried out at 60°C for 30 min and then terminated by 0.25 mL of 0.1 M Na_2_CO_3_ added. The mixture was centrifuged at 10,000 rpm for 15 min and the absorbance was determined at 410 nm using spectrophotometer (spectrophotometer: Lambda EZ 201 Perkin Elmer, USA). One unit of lipase activity was defined as the amount of enzyme that caused the release of 1 *μ*mol of* p*-nitrophenol (molar absorption coefficient 4.6 mM^−1^ cm^−1^) from* p*NP-laurate in 30 min under test conditions [18, 19].

### 2.5. Determination of Protein Content

Protein content was determined according to Lowry Method [20].

### 2.6. Optimization of Various Production Conditions of TA Lipase from* G. thermoleovorans* DA2

Several experiments were conducted to study the effect of physical and nutrients on culture conditions for TA lipase production by* G. thermoleovorans* DA2. All the previously mentioned production conditions were investigated throughout the following experiments.

#### 2.6.1. Effect of Incubation Temperature on Production of TA Lipase from* G. thermoleovorans* DA2

Most favorable production temperature was studied by incubating the inoculated production medium at varying temperatures (50, 55, 60, 65, 70, 75, 80, 85, and 90°C). The culture filtrate was used for the lipase activity.

#### 2.6.2. Effect of pH of the Medium on the Production of TA Lipase from* G. thermoleovorans* DA2

For optimization of production pH, the production medium of different pH, namely, 6, 7, 8, 9, 10, 11, and 12, was inoculated with culture and incubated in shaker for 18 hrs at 65°C and lipase activity of culture filtrate was determined.

#### 2.6.3. Effect of Incubation Time on Production of TA Lipase from* G. thermoleovorans* DA2

To optimize incubation time for the maximum production of lipase, the production medium was incubated at 60°C in the shaker for 12, 18, 24, 36, 48, 56, and 72 hrs.

#### 2.6.4. Effect of Carbon Source on Production of Lipase from* G. thermoleovorans* DA2

Various carbon sources such as glucose, galactose, xylose, ribose, rhamnose, melezitose, sucrose, lactose, maltose, cellobiose, sorbitol, mannitol, and inulin were used in the production medium at the concentration of 1% (w/v) to check the effect of carbon source on lipase production. The medium without carbon source served as control and the culture filtrate was assayed for enzyme activity.

#### 2.6.5. Effect of Nitrogen Source on Production of TA Lipase from* G. thermoleovorans* DA2

To study the effect of nitrogen source, various nitrogen sources such as ammonium chloride, ammonium sulphate, ammonium nitrate, ammonium molybdate, ammonium phosphate, sodium nitrite, urea, calcium nitrate, potassium nitrate, and peptone were conducted in the production medium at the concentration of 0.5% (w/v). The main medium included sodium nitrate only served as control. The culture filtrate was assayed for enzyme activity.

#### 2.6.6. Effect of Substrate Concentration on Production of Lipase from* G. thermoleovorans* DA2

The effect of substrate concentration on the production of TA lipase was determined by varying the concentration of fatty restaurant wastes, that is, 0.25, 0.5, 1, 2, 5, 10, 20, and 40% (w/v).

#### 2.6.7. Effect of Inoculum Size on Production of Lipase from* G. thermoleovorans* DA2

Heavy cell suspension of* G. thermoleovorans* DA2 was prepared by growing bacterial isolate on nutrient broth and limiting growth level at 3 × 10^7^ CFU·mL^−1^. Different inocula sizes of culture including 0.25, 0.5, 1, 2, 4, 5, and 10% (v/v) were applied.

#### 2.6.8. Effect of Agitation Rate on Production of TA Lipase from* G. thermoleovorans* DA2

To optimize the agitation rate for maximum lipase production, the inoculated production medium was agitated at different rotations per minute (rpm) such as 40, 80, 120, and 150 rpm. The culture filtrate was used to check the enzyme activity.

### 2.7. Application of TA Lipase from* G. thermoleovorans* DA2 in Degreasing of Leather

The crude TA lipase from* G. thermoleovorans* DA2 was used as degreaser agent in leather industry as compared to traditional methods which depends upon solvents and surfactants.

#### 2.7.1. Preparation of Skin Samples

A piece of sheep skin was obtained after it had undergone the common processing steps, that is, liming, dehairing, and bating. Eight skin pieces about 5 × 5 cm dimension were cut and divided into 4 groups (each of 2 pieces):

 Group (A): the skin was soaked in 50 mL (Kerosene) and was incubated at 25°C as a traditional method.
 Group (B): the skin was immersed in 50 mL of 10% of the crude TA lipase and was incubated at 60°C.
 Group (C): the skin was treated with a mixture of crude TA lipase (10%) + Kerosene in 4 : 1 ratio and was incubated at 60°C.
 Group (D): the skin was bathed in a mixture of crude TA lipase (10%) + Triton X-100 in 10 : 1 ratios and was incubated at 60°C.

All groups of skin were treated for 2 hrs then; the skin was collected and dried [21].

#### 2.7.2. Total Lipid Measurement Technique

Total lipid of the treated skin pieces were measured relying on Soxhlet apparatus [22]. The obtained values were then compared with the lipid content of the control.

## 3. Results

### 3.1. Bacterial Strain Selection and Identification

Out of seven isolates the most efficient lipase producer was isolated from desert of southern Sinai based on enzyme assay method and it was identified using 16S rRNA and selected for further studies.

### 3.2. Bacterial Identification by 16S rRNA

The amplified 16S rRNA gene fragment was investigated using DNA ladder (Gene ruler 50 bp–1031 bp DNA ladder) and it was 380 bp.

**Figure 1:**
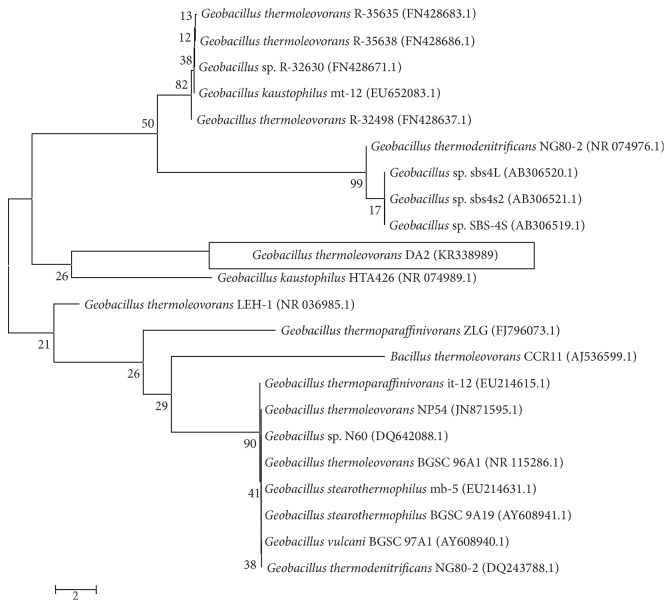
Phylogenetic position of* Geobacillus thermoleovorans* DA2 within the genus* Geobacillus*. The branching pattern was generated by neighbor-joining tree method and the GenBank accession numbers of the 16S rRNA nucleotide sequences are indicated in brackets. The bar indicates a Jukes-Cantor distance of 2.

The BLAST algorithm was used to retrieve for homologous sequences in GenBank to the obtained 16S rRNA sequence. The bacterial isolate revealed 99% identity to full genome of* Geobacillus thermoleovorans*,* Geobacillus stearothermophilus*,* G. thermoparaffinivorans*,* G. thermodenitrificans*, and* G. kaustophilus*. Based on the morphological, biochemical, and molecular characteristics, the isolate was identified and released in NCBI GenBank as* Geobacillus thermoleovorans* DA2 under the accession numbers (KR338990), and the branching pattern was analyzed by 500 bootstrap replicates as in Figure 1.* G. thermoleovorans* DA2 is an aerobic, spore-forming, nonmotile rod able to grow at high temperatures (50–80°C) with an optimum growth at 65°C.

### 3.3. Optimization of Production Conditions of TA Lipase from* G. thermoleovorans* DA2

#### 3.3.1. Effect of Incubation Temperature on Production of TA Lipase from* G. thermoleovorans* DA2

**Figure 2:**
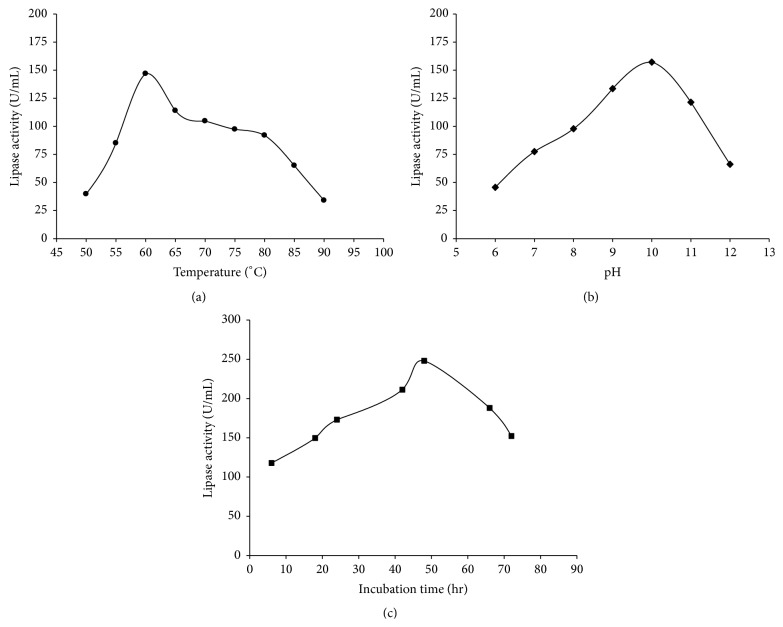
(a) Effect of incubation temperature on production of TA lipase from* G. thermoleovorans* DA2. (b) Effect of pH on production of TA lipase from* G. thermoleovorans* DA2. (c) Effect of incubation time on production of TA lipase from* G. thermoleovorans* DA2.

Most suitable temperature for maximum production of TA lipase (146.85 U/mL) from* G. thermoleovorans* DA2 was found to be 60°C as shown in Figure 2(a).

#### 3.3.2. Effect of pH of the Medium on Production of TA Lipase from* G. thermoleovorans* DA2

The maximum production of TA lipase from* G. thermoleovorans* DA2 was observed at pH 10 (157.15 U/mL) and after that, the lipase activity was decreased with increasing the pH values as shown in Figure 2(b).

#### 3.3.3. Optimization of Incubation Time for Production of TA Lipase from* G. thermoleovorans* DA2

The maximum production of lipase was observed at 48 hrs (248.11 U/mL). Optimal incubation time was found to be 48 hrs corresponding to maximum enzyme activity and after this decline in enzyme activity was observed (Figure 2(c)).

#### 3.3.4. Effect of Carbon Source on Production of TA Lipase from* G. thermoleovorans* DA2

**Figure 3:**
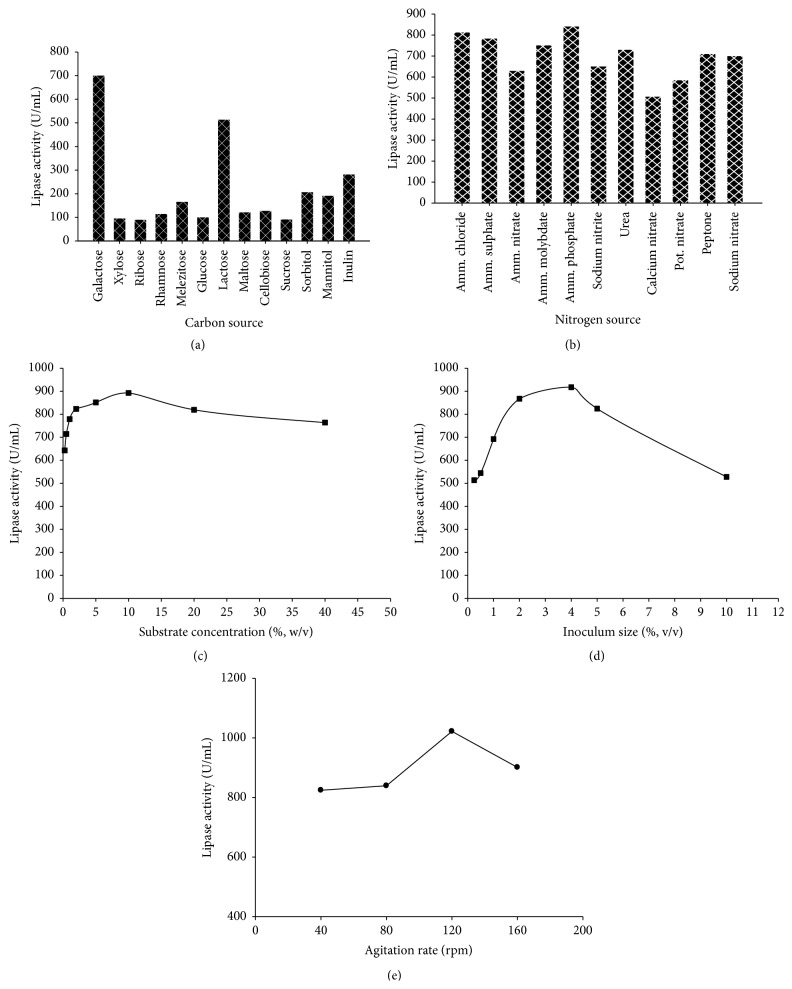
(a) Effect of carbon source on production of lipase from* G. thermoleovorans* DA2. (b) Effect of nitrogen source on production of lipase from* G. thermoleovorans* DA2. (c) Effect of substrate concentration on production of TA lipase from* G. thermoleovorans* DA2. (d) Effect of inoculum size on production of TA lipase from* G. thermoleovorans* DA2. (e) Effect of agitation rate on production of lipase from* Geobacillus thermoleovorans* DA2.

Maximum production of TA lipase was obtained from the medium which was supplemented with galactose (1%) as carbon source giving enzyme activity of 701.86 U/mL (Figure 3(a)).

#### 3.3.5. Effect of Nitrogen Source on Production of TA Lipase from* G. thermoleovorans* DA2

The lipase production was highest (843.04 U/mL) in medium containing ammonium phosphate at a concentration (0.5%) as nitrogen source (Figure 3(b)).

#### 3.3.6. Effect of Substrate Concentration on Production of TA Lipase from* G. thermoleovorans* DA2

Maximum enzyme activity (892.43 U/mL) was observed with 10% (w/v) fatty restaurant wastes (Figure 3(c)).

#### 3.3.7. Optimization of Inoculum Size for Production of TA Lipase from* G. thermoleovorans* DA2

Figure 3(d) reveals that the inoculum size (4%, v/v) gave the maximum lipase production with an activity of 917.23 U/mL.

#### 3.3.8. Effect of Agitation Rate on Production of TA Lipase from* G. thermoleovorans* DA2

The maximum lipase activity (1021.91 U/mL) was observed at 120 rpm as shown in Figure 3(e).

### 3.4. Application of TA Lipase in Degreasing of Leather

The thermoalkaliphilic lipase from* G. thermoleovorans* DA2 plus Triton X-100 was the most efficient leather degreasing agent where the total lipid content decreased from 17.5% to 2.6% as shown in Table 1.

The mixture of TA lipase plus kerosene decreased the total lipid content to 5.7%; so it came in second level.

## 4. Discussion

The applications of lipases are constantly increased in industrial and biotechnological fields; therefore, that should be supported by discovering novel lipase types with improved characters. The common about enzymes is their sensitivity to adverse conditions such as high temperature, extreme acidity and/or alkalinity, drought, and high salinity, but extremozymes, enzymes derived from extremophilic microorganisms, are an attractive alternative to tuning a given biocatalyst for a specific industrial application. They are capable of catalyzing their respective reactions in nonaqueous environments, water/solvent mixtures, at extremely high pressures, acidic and alkaline pH, at temperatures up to 140°C, or near the freezing point of water [23]. The present investigation is an attempt to discover novel bacterial strains with the capability of producing lipases able to tolerate high temperature and high alkalinity as well. Seven of twenty three strains showed different lipolytic activities in incubation temperature 65°C and pH 9; the most potent producing strain is identified as* G. thermoleovorans* DA2 selected to further studies as the most promising source of thermoalkaliphilic lipase.

Optimum temperature in the present investigation came in correspondence with the optimum temperature for lipase produced by* G. stearothermophilus* strain-5 [24]. On the other hand, the temperature for the highest production of lipase by* G. thermodenitrificans* AZ1 was 55°C [25].

Optimum pH value for the present study suggests more alkaliphilic behavior comparing to lipases produced by* Geobacillus thermoleovorans* CCR11 and the alkaliphilic* Bacillus* sp. (KS4) which was found to give their highest lipase productions at pH 8 [26, 27]. Berekaa et al. reported that the optimum pH for lipase production by* G. stearothermophilus* strain-5 was 7 [28].

*G. thermoleovorans* DA2 was found to consume 48 hrs to give its maximum production of TA lipase comparing to* Bacillus thermoleovorans* CCR11 which is recorded to produce the highest lipase rate after 44 hrs, while the highest production of lipase by the thermophilic strain* Bacillus* sp. strain 42 was recorded after 72 hrs [26, 29].

Galactose was found to be the most supportive carbon source for the TA lipase production by* G. thermoleovorans* DA2, suggesting high ability of the strain to uptake and utilize the monosaccharide galactose in the metabolic processes of lipase production as the lactose disaccharide was the second optimum carbon source. Glycerol was found to be the most inducing carbon source for lipase production by* G. stearothermophilus* strain-5, while it is the highest lipase production by* B. subtilis* ZR-1 supported by the sole olive oil [12, 28].

Peptone was the most supportive nitrogen source for lipase production by* Bacillus* sp. LBN2 [30]. In contrast, mix of yeast extract and peptone was the best for the production of thermoalkaliphilic lipase by* B. coagulans* BTS-3 [31]. Moreover, sole yeast extract was the best for lipase produced by* B. licheniformis* MTCC-10498 [32].

Abd El Rahman found that 12.5% of the slaughter house waste was the optimum substrate concentration for lipase production by* B. stearothermophilus* B-78, while Gayathri et al. have reported that 2% of palm oil was the optimum concentration for lipase production by* B. stearothermophilus* [33, 34].

Ten percent (10%, v/v) was the optimum inoculum for lipase production by* B. licheniformis* MTCC-10498 [32]. However,* Bacillus flexus* XJU-1 gave the best production of lipase depending on 2% (v/v) inoculum size, while 6% (v/v) was the optimum inoculum size for the best lipase production by* Pseudomonas gessardii* [35, 36].

The optimum revolution per minute for the maximum production of TA lipase by* G. thermoleovorans* DA2 was found to be 120 rpm, which came in complete accordance with the optimum agitation rate for highest lipase production by* B. licheniformis* MTCC-10498 [32], while Veerapagu et al. reported that the optimized value for greatest lipase production by* Pseudomonas gessardii* was 160 rpm [36].

Once samples of sheep leather were treated with TA lipase produced by the strain* G. thermoleovorans* DA2 mixed with Triton X-100, fatty content of the leather depleted from 17.5% to 2.5%. This may be due to the elevated processing temperature which takes a hand in destroying the walls of skin fat cells and increase solubility of the fat content very well. Moreover, Triton X-100 had supportive effect in reducing the surface tension of the lipids and facilitated the enzyme efficacy. Saran et al. recorded degreasing rate more than 95% by 5–10% mesophilic lipase enzyme produced by* Bacillus subtilis* solely after treating for 12 hrs [11].

Moreover, their interpretations came in complete accordance with our observations about the higher quality, appearance, and smoothness levels of the enzyme-treated leather compared to chemically processed skin. The obtained results are suggesting that advanced efforts to replace the ecohazardous traditional chemical tanning process with the green enzymatic techniques are worthy to be achieved.

## 5. Conclusion

In the present study, thermoalkaliphilic lipase was produced from the new bacterial isolate which was identified using the sequence of 16S rRNA gene and biochemical tests as* Geobacillus thermoleovorans* DA2. Wide ranges of growth factors were studied for maximizing TA lipase enzyme. The production of TA lipase with high amount using cheap substrate (fatty restaurant wastes) made the production process cost-effective. The results exhibited the potential application of the TA lipase in leather industry as a degreaser suggesting more ecofriendly tanning processes in the future.

## Figures and Tables

**Table 1:** Effect of using the TA lipase from *G. thermoleovorans *DA2 as a degreasing agent and the total lipid content of leather samples.

Group of leather samples	Type of leather treatment	Total lipid content (%)
	Control (without treatment)	17.50
Group (A)	Organic solvent (Kerosene)	7.50
Group (B)	10% crude lipase produced by* G. thermoleovorans* DA2	8.90
Group (C)	10% crude lipase produced by* G. thermoleovorans* DA2 + Kerosene	5.70
Group (D)	10% crude lipase produced by* G. thermoleovorans* DA2 + Triton X-100	2.60
